# Probabilistic and Deterministic Assessment of Dietary Iron Intake for Armenian Adult Population Using Individual and Household-Based Approaches

**DOI:** 10.3390/foods15213868

**Published:** 2026-10-29

**Authors:** Davit Pipoyan, Meline Beglaryan, Bagrat Harutyunyan, Yepraqsya Arshakyan, Taron Kareyan, Ani Asatryan, Lilit Sahakyan

**Affiliations:** Center for Ecological-Noosphere Studies (CENS), Yerevan 0025, Armenia; david.pipoyan@cens.am (D.P.); bagrat.harutyunyan@cens.am (B.H.); yepraqsya.arshakyan@cens.am (Y.A.); taron.kareyan@cens.am (T.K.); ani.asatryan@cens.am (A.A.); lilit.sahakyan@cens.am (L.S.)

**Keywords:** diet, dietary reference value, micronutrient, daily intake, Monte Carlo simulation, 24 h dietary recall, household budget survey

## Abstract

The study aimed to estimate dietary iron intake in Armenia by integrating individual-based probabilistic modelling with household-based deterministic assessment. Iron contents were determined in 146 composite samples prepared following a Total Diet Study framework and analysed with ICP-OES. Individual food consumption data from a national single 24 h dietary recall survey of 1400 adults were combined with determined iron contents, and Monte Carlo simulation characterized the distributions of observed one-day iron intake among men, women aged 18–39 years and women aged ≥40 years. In addition, household food consumption data from the Armenian Integrated Living Conditions Survey (2019–2024) were used for deterministic assessment of population-level trends and major dietary contributions. Mean total dietary iron intake estimated by probabilistic modelling was 10.39, 8.31 and 8.26 mg/day for men, women aged 18–39 years and women aged ≥40 years, respectively. Non-heme iron had the major share in total iron intake across all groups. Mean and median intakes exceeded the EFSA Average Requirement (AR) but remained below the Population Reference Intake (PRI), particularly among women of reproductive age. Deterministic assessment showed that per capita dietary iron intake of the general population varied from 8.07 to 9.27 mg/day during 2019–2024, with flour-based products as the main contributors. Overall, the integrated assessment framework provides a comprehensive characterization of dietary iron intake in Armenia and supports the development of targeted nutrition strategies addressing potentially inadequate dietary iron intake among vulnerable population groups, while biomarker data are required to evaluate iron status.

## 1. Introduction

Iron (Fe) is an essential micronutrient involved in oxygen transport, electron transfer, oxidase activity, energy metabolism and DNA synthesis. Because of its important physiological roles, inadequate iron intake or impaired iron status can lead to a range of adverse health outcomes [1,2]. Among these, iron deficiency is one of the most prevalent nutritional disorders and leading causes of anaemia worldwide, primarily affecting women of reproductive age and children [3,4,5]. The World Health Organization (WHO) recognizes iron deficiency anaemia (IDA) as a major public health problem, with substantial economic and social consequences [2,6]. Despite progress over recent decades, IDA continues to represent a significant global health burden, and most low- and middle-income countries are unlikely to achieve the WHO Global Nutrition Target of reducing anaemia by 50% among women of reproductive age by 2030. Recent geospatial analyses have identified the Caucasus and Central Asia as a region of persistent nutritional vulnerability [7,8]. Hence, strengthening the evidence base on dietary iron intake remains an important priority for countries in these regions, including Armenia.

In Armenia, iron nutrition reflects the complexity of transitional food systems and dietary patterns. Among women of reproductive age, anaemia prevalence remains around 17%, with sub-national variation (7–39%). This burden coexists with high prevalence of food insecurity, low meat consumption, and a dietary pattern in which iron-rich plant-based foods are subject to seasonal fluctuations in availability and intake [9,10,11]. Such dietary characteristics affect not only total iron intake but also its nutritional effectiveness, as iron bioavailability depends on its chemical form (i.e., heme and non-heme) and overall dietary composition. Iron is present in foods as heme iron, found mainly in animal tissues and characterized by higher bioavailability (around 25%), and non-heme iron, which predominates in plant foods, dairy, eggs and iron-fortified products. Hence, two diets providing the same total iron intake can result in different levels of absorbed iron [12,13,14]. So, even when dietary intakes meet dietary reference values (DRVs), iron status may remain inadequate, contributing to a persistent risk of IDA. This phenomenon has been documented in populations with plant-based and cereal-dominant diets in low- and middle-income countries [15,16]. In the typical Armenian diet, inhibitors of non-heme iron absorption, including phytates in cereals and legumes, polyphenols and tannins in tea and coffee, coexist with relatively few enhancers, notably ascorbic acid from fruits and vegetables and the so-called “meat factor” associated with muscle proteins [17,18,19].

Previous deterministic assessment of dietary iron intake among males and females (including women of reproductive age) indicated that a substantial proportion of the Armenian adult population does not meet recommended intake levels [17]. However, these estimates are constrained by methodological limitations. First, dietary intake was assessed using a single 24 h recall, which does not adequately capture intake distributions or seasonal and inter-annual variability in food consumption patterns [17,20,21]. Second, iron content data were derived from a limited number of samples analysed using flame atomic absorption spectrophotometry (AAS) [22]. These study-specific limitations highlight the need for improved dietary assessment frameworks that integrate both individual-based and household-based approaches. Although nationally representative individual dietary surveys are considered the preferred approach for estimating nutrient intake, they are resource-intensive and conducted infrequently in many countries. In contrast, household budget surveys (HBSs) remain one of the most widely available sources of food consumption data across the Caucasus, including Armenia, and other comparable middle-income settings [23]. While household-based assessments have some limitations (e.g., inability to capture food waste and out-of-home food consumption), they remain an important practical tool for monitoring nutrient intake trends in countries where nationally representative individual dietary surveys are unavailable or not regularly conducted [24,25,26]. In this context, the 24 h recall dataset [17,18,19] remains the only nationwide individual-based food consumption dataset available for the Armenian adult population. It therefore can be used to characterize observed one-day dietary intake.

In addition to the choice of dietary survey, nutrient estimates are influenced by the applied methodological frameworks. Deterministic methods based on point estimates of food consumption and nutrient contents are very useful screening tools but may inadequately characterize variability and uncertainty within populations. In contrast, probabilistic methods, including observed individual means and Monte Carlo simulation, can incorporate variability in consumption data and, where replicate or distributional data are available, in content data, thereby providing a more realistic characterization of intake distributions, particularly at the lower and upper tails of the distribution, which are critical for nutritional assessments [22,27,28]. Recent developments in dietary intake assessments have therefore emphasized the complementary application of deterministic and probabilistic methods to improve the characterization of population intake distributions and strengthen the evidence base for nutrition policy and risk-based decision-making [29]. These methodological considerations are particularly relevant for iron.

To address these challenges of regional and international relevance, the present study was conducted within the framework of the State Targeted Program “Scientific and Methodological Support for Micronutrient Intake Assessment and Public Awareness in the Republic of Armenia”, with the aim of strengthening the evidence base for dietary iron intake assessment in the country. The study characterized population-level dietary iron intake by integrating a deterministic household-based scenario with a probabilistic individual-based Monte Carlo modelling framework. This integrated approach is intended to improve the robustness of nutrient intake assessment in a data-limited setting and supports evidence-based, country-specific iron intervention strategies.

## 2. Materials and Methods

### 2.1. Iron (Fe) Analysis in Food Samples

Food sampling and analysis were carried out within the framework of the State Target Program “Scientific and Methodological Support for Micronutrient Intake Assessment and Public Awareness in the Republic of Armenia” (2025). A sampling plan was prepared for foods most consumed by the Armenian population and considered as potential sources of dietary iron. A list of food items to be studied was prepared, and target food groups were selected based on available food consumption information, emphasizing relevant databases compiled by the Center for Ecological-Noosphere Studies (CENS) [17,19] and data from the Statistical Committee of the Republic of Armenia [30].

Food sampling and sample pre-treatment were done following a Total Diet Study (TDS) approach. Unlike surveillance and monitoring procedures, in which food items undergo analysis in raw condition, one of the major features of the TDS method is that, prior to analyses, food stuffs undergo culinary treatment to replicate the preparation methods that consumers commonly use [31]. The TDS approach included the following procedures: selection and sampling of food assortment (a food item is sampled in the same manner it is purchased by a consumer); culinary treatment of raw food items; aggregation of food items into representative food groups; homogenization of composite samples and determination of iron contents in food samples.

Prior to laboratory analysis, food subsamples underwent relevant treatment (removal of non-edible parts, cooking, frying, etc.). Then the treated subsamples were mixed to obtain a single composite sample per food item. In total, over 382 subsamples of foods consumed in Armenia underwent pre-treatment in compliance with local food habits. The prepared subsamples were then combined to form composite representative food samples. Finally, 146 composite samples of food were prepared as intended for iron analyses.

Before iron analysis, the composite samples were homogenized at CENS’s analytical laboratory by a homogenizer with stainless steel knives, which was washed with soapy water, tap water and distilled water after each sample and then dried and prepared to homogenize a subsequent sample [32].

Acid digestion of samples: approximately 0.5-2.0 g of each homogenized sample was accurately weighed using an analytical balance (Shimadzu AP224W, Kyoto, Japan; readability 0.0001 g). The samples were digested in a microwave digestion system (ETHOS EASY, Milestone, Sorisole, Italy) according to GOST EN 14084-2014 [33] using 5.0 mL of HNO_3_ (65%, Carlo Erba Reagents, RS-Ultrapure grade) and 2.0 mL of H_2_O_2_ (30%, Carlo Erba Reagents, RS-Ultrapure grade). The microwave digestion (Milestone ETHOS EASY microwave digestion system with MAXI-44 rotor, Milestone, Sorisole, Bergamo, Italy) procedure was performed in accordance with US EPA Method 3051A (2007) [34].

After digestion, the vessels were allowed to cool to room temperature (20 °C). The digests were quantitatively transferred into 50 mL polypropylene volumetric flasks and diluted to volume with deionized water (18 MΩ·cm, ADRONA SIA CB-1903, Riga, Latvia). The resulting solutions were filtered through 0.45 μm membrane filters using a vacuum filtration system (STERIFIL, Merck Millipore, Burlington, MA, USA) according to GOST EN 14084-2014 [33]. The same procedure was applied to reagent blanks, certified reference materials (BCR-679 White Cabbage (Elements) (European Commission, Joint Reserach Centre, Geel, Belgium) and NCS ZC73031 Carrot (China National Analysis Center for Iron and Steel, Beijing, China)), and matrix spike samples for quality assurance and quality control (QA/QC).

Iron analysis: elemental analysis was performed using an iCAP PRO ICP-OES spectrometer (Thermo Fisher Scientific, Monza, Italy), with high-purity argon (99.9999%) as the plasma gas. Iron was determined at the wavelength of 259.940 nm (Aqueous-Radial-iFR). Instrument operating conditions were established according to the Thermo Scientific iCAP 7400 ICP-OES Duo Application Note 40755 [35].

Iron concentrations in food samples were determined based on calibration curves obtained from multi elemental standard solution (100 mg-L^−1^) (Carlo Erba Reagents, Milan, Italy; Cat. No. 504311). Calibration curves were constructed using eight concentration levels, ranging from 0.01 to 15 mg L^−1^. All standards were prepared by serial dilution with 3% HNO_3_ (Carlo Erba Reagents, RS-Ultrapure grade) [35].

Quality Assurance and Quality Control (QA/QC): QA and QC procedures were performed in accordance with ISO/IEC 17025 requirements [36]. Linearity of the calibration curves for the method was observed in the concentration range 0.01–15 mg/L, with coefficients of determination (R^2^) exceeding 0.999 for all analytes. The sensitivity of the method was characterized by the Limit of Detection (LOD = 0.0077 mg/kg) and Limit of Quantification (LOQ = 0.0230 mg/kg), which were calculated from the slope of the calibration curve and the standard deviation of a blank sample. Reagent blanks were carried through the complete digestion procedure with each analytical batch, and iron was not detected in any of them. Therefore, blank correction was not required.

The accuracy and reproducibility of the analysis were assessed using the SRMs (BCR-679 and NCS ZC73031) and a spike sample containing a defined quantity of a standard solution. Recovery was assessed as follows. Each composite sample was digested and measured with three replicate injections, the reported concentrations being the mean of these readings. For the SRM “White cabbage (elements)” (BCR-679), three independent determinations gave a mean measured content of 45.7 ± 6.62 mg/kg against a certified value of 55 mg/kg, corresponding to a mean recovery of 83.1 ± 12.0% (range 74.4–96.8%), whereas for the SRM “Carrot” (NCS ZC73031), two determinations gave 110.58 ± 7.32 mg/kg against a certified value of 148 mg/kg, i.e., a mean recovery of 74.7 ± 4.9% (range 71.2–78.2%). In eight food samples fortified with 0.05 mg Fe each, the mean recovery was 98.6 ± 7.5% (range 88.4–111.2%). The detailed quality control results are presented in Supplementary Tables S14 and S15.

### 2.2. Daily Fe Intake Assessment

Dietary iron intake *(DI)* was estimated using the following equation:

(1) $$DI\;=\;C_{Fe}\;\times \;C_{food}$$

where *DI* is the estimated daily intake of iron (mg/day); *C_Fe_* is the iron content in each food item (mg/kg, converted from mg/100 g), and *C_food_* is the daily consumption of that food item (kg/day). For *C_Fe_*, results below the LOD were handled using the lower-bound approach, i.e., substituted with zero. Due to the low LOD and the small number of results below the LOD (4 samples out of 146), the choice between lower-bound (zero), middle-bound (LOD/2) and upper-bound (LOD) substitution did not considerably affect the estimated daily iron intakes.

The DI was further estimated separately for the two basic forms of dietary iron, namely heme (iron from animal-origin sources such as meat, meat products, and fish) and non-heme (iron mainly from plant-origin sources). Iron in eggs, milk and dairy products, and in all plant-origin foods was considered entirely non-heme. Following the approach used in similar dietary studies [17,37], heme iron was assumed to represent 40% of total iron in meat, meat products, offal, poultry and fish, as well as meat-containing composite dishes, with the remaining 60% being non-heme iron. This fixed 40/60% split is a population-level simplification, as the proportion of heme iron may vary among different types of meat, meat-containing products and composite dishes [17]. For each individual, heme and non-heme iron intakes were calculated from the same reported foods, so that at the individual level total iron intake is the sum of heme and non-heme iron intakes. It should be noted that the summary statistics are reported for different population bases: total and non-heme iron intakes refer to all participants of each group, whereas heme iron intakes refer only to those participants who reported at least one heme iron source (i.e., meat, meat products, poultry, offal, fish or meat-containing composite dishes) on the recall day. Consequently, the group means of heme and non-heme iron are not additive, and their sum exceeds the group mean of total iron intake.

Dietary iron intakes were assessed using two complementary methodological approaches based on different food consumption databases. First, an individual food consumption database derived from the first national 24 h dietary recall survey of adults (n = 1400 adults, including men, n = 666; and women, n = 734) [17,19] was used to carry out the probabilistic assessment of dietary iron intake. Full methodological details of this dietary survey are presented in our earlier work based on the same database [17,19]; the features relevant to the present assessment are outlined below. Fieldwork spanned from February to September 2021 and covered residents aged 18 years and over in every region (Armavir, Ararat, Aragatsotn, Gegharkunik, Kotayk, Lori, Shirak, Syunik, Tavush, Vayots Dzor) of the country and in the capital city of Yerevan. The participants were recruited according to quotas based on the adult population distribution by region, sex and age group. The recruitment continued within each stratum until its target was reached. The sample included 822 respondents from the regions and 578 from Yerevan, with the Yerevan quota distributed across all 12 administrative districts. The respondents ranged in age from 18 years to 80 years and above. Interviewers trained for this task conducted the recalls in person or by telephone, working from a standardized form on which everything eaten and drunk on the preceding day was recorded in quantitative terms. The consumption database comprises 164 items (124 single food items and 40 composite dishes) [19]. The iron contents determined in this study were matched with these food consumption data, i.e., the iron concentration measured in each analysed composite sample was assigned to the corresponding food item reported in the 24 h dietary recall survey, and the iron concentrations determined for the analysed composite dishes were assigned to the same dishes reported by the respondents. Second, household food consumption data derived from the “Household’s Integrated Living Conditions Survey (ILCS) anonymized microdata database (purchased and consumed food and non-food products)” of the Statistical Committee of the Republic of Armenia (ARMSTAT) [30] were used to carry out the deterministic assessment of dietary iron intake.

Probabilistic assessment of dietary iron intake: for the probabilistic assessment, individual dietary intake was estimated by combining data on each serving of each food item eaten within 24 h and iron contents of the food items. Dietary iron intake was assessed separately for men and for women according to the groups used by EFSA for adult iron Dietary Reference Values (DRVs) [1], i.e., premenopausal women aged 18–39 years and postmenopausal women aged ≥40 years. As menopausal status was not among the variables recorded in the 24 h dietary recall survey, women aged 18–39 years (n = 313) were considered within the premenopausal group, while women aged ≥40 years (n = 421) were considered within the postmenopausal group. This grouping was applied to align the study population with the age- and sex-specific categories used by EFSA for establishing iron DRVs. Because menopausal status was not verified, the two groups of women are referred to throughout the manuscript by age, i.e., women aged 18–39 years and women aged ≥40 years, while the EFSA DRVs established for premenopausal and postmenopausal women, respectively, were applied to them. The individual food consumption database enabled us to compute the daily iron intake for each consumer surveyed. Estimated daily dietary iron intake was calculated for each surveyed individual by summing the iron contribution from all reported food items. Reported intakes were screened for implausible values using the interquartile range (IQR) criterion. The flagged reports corresponded to plausible high consumption of staple foods rather than to reporting errors and were therefore retained, so that all 1400 records were included in the analysis. Data handling was performed with IBM SPSS Statistics version 22. When using IBM SPSS Statistics, mathematical operations were all performed employing a “Compute Variable” tool(IBM SPSS Statistics, version 22). Additionally, data for different age groups were separated using a “Recode into Same Variables” tool. To split the data obtained into groups and make data representation more detailed, the tool “Split File” was used. To characterize the distribution of observed one-day dietary iron intake within the study population, a probabilistic Monte Carlo simulation (with 100,000 iterations) was applied using @RISK (Lumivero’s Decision Tools Suite). The simulation outputs included distributions of total dietary iron intake, as well as heme and non-heme iron intake separately, for men, women aged 18–39 years and women aged ≥40 years. Because iron content was determined from a single composite sample for each food item, the variability in daily dietary iron intake was represented through the individual food consumption data. The @Risk “Fit” tool was used to identify and assign appropriate probability distributions to the food consumption data, which were then used as model inputs. The corresponding calculated daily intake data were fitted separately and used as model outputs. The first best-fitted distribution recommended by the “Fit” tool was selected for the respective datasets: a PERT distribution for total iron and non-heme iron intake and a Triangular (Triang) distribution for heme iron intake. It is important to note that the distribution of heme iron intake was fitted only to the records of participants who reported at least one heme iron source on the recall day. Since total, non-heme and heme iron intake were fitted separately and with different distributions, the summary statistics of the three simulated outputs are not additive; hence, the simulated mean heme and mean non-heme iron intakes do not sum to the simulated mean total iron intake. The variables included in the model, the fitted distributions, and the corresponding goodness-of-fit statistics are summarized in Supplementary Table S16.

Deterministic assessment of dietary iron intake: for the deterministic assessment, the daily iron intake was calculated by multiplying the iron content of food by its average daily household consumption from ILCS for the period 2019–2024 [30]. The ILCS is an annual nationally representative household survey conducted by the ARMSTAT, in which households record the quantities of food purchased and consumed. Therefore, the deterministic estimates refer to the average per capita intake of the general population. No adult-equivalent adjustment was applied, so the deterministic estimates represent the average intake of a household member of any age, including children and the elderly, whose food and energy intake are lower than those of adults. Therefore, the deterministic estimates are expected to underestimate the intake of adults, and their comparison with the EFSA DRVs for adults is indicative rather than quantitative. The individual-based probabilistic estimates remain the basis for assessing the adequacy of an adult’s dietary intake of iron. In addition, ILCS quantities refer to foods acquired by the household, whereas the iron contents determined in this study refer to foods as consumed. For each food item, the analysed composite sample corresponded to the form in which the food is normally consumed in Armenia, and no edible-portion factors were applied to the acquired quantities; therefore, any difference between the acquired weight and the edible, as-consumed weight remains a source of uncertainty in the deterministic estimates. Since the same analytical iron concentrations (2025 sampling) were applied to all survey years, inter-annual changes in the deterministic estimates reflect changes in the reported quantities of foods only. Food groups and the lists of food items contained in each group were compiled using the Statistical Committee’s food classification approach. Total daily iron intake was calculated as the sum of iron intakes from all food items included in the assessment. Quantitative calculations, including mean iron contents, estimated daily intake from each food group, and total dietary iron intake, as well as the share of food groups in daily iron intake, were performed using Microsoft Excel. Graphic representation of the results was also prepared using Microsoft Excel.

## 3. Results

### 3.1. Fe Content in Armenian Diet

Iron (Fe) contents determined in the studied 146 composite food samples representative of the Armenian diet are presented in the Supplementary Material (Tables S1–S11). As shown by our research results, iron contents varied notably among the studied foods and food groups. Among fruits commonly consumed in the Armenian diet, fresh fruits generally contained low iron contents. Iron contents in apples, peaches, oranges and grapefruits were below the LOD, whereas dried fruits contained relatively higher iron contents than their fresh counterparts. A similar pattern was observed for herbs, with dried herbs (spices) consistently having higher iron content than fresh herbs (mixed greens including coriander, parsley, basil and dill). From the studied vegetables, processed products generally contained higher iron contents than fresh vegetables, particularly tinned grape leaves, tomato paste, and tinned green peas. Within flour-based foods included in the Armenian diet, iron contents differed according to flour type and the degree of milling. Of the studied kinds of bread, relatively high in iron were wholegrain and fitness breads. In the group of bakery products, sources of iron included waffles and cookies (especially those containing chocolate or cocoa). Animal-origin foods also showed considerable differences in iron contents. Meat and meat products generally contained higher iron contents, whereas dairy products had substantially lower iron contents (0.9–7.2 mg/kg). The ten food products with the highest iron contents among plant-origin and animal-origin foods are presented in Figure 1 and Figure 2, respectively.

The plant-origin foods with the highest iron contents (Figure 1) were dried basil (≈506 mg/kg), bitter chocolate (≈123 mg/kg), ground black pepper (≈103 mg/kg), dried plums (≈71 mg/kg), dill (≈58 mg/kg), mixed greens (≈57 mg/kg), almonds (≈51 mg/kg), sunflower seeds (≈49 mg/kg), tinned grape leaves (≈47 mg/kg) and cilantro (coriander) (≈45 mg/kg).

The animal-origin products (and animal product-based foodstuffs) with the highest iron contents (Figure 2) were offal, known as “tzhvzhik” in Armenia (≈125 mg/kg), basturma and sudzhukh, traditional cured meat products (≈64 mg/kg), beef (≈43 mg/kg), beef liver (≈38 mg/kg), hot dogs (≈28 mg/kg), fried eggs (≈24 mg/kg), mutton and lamb (≈23 mg/kg), grape leaf tolma, a traditional minced meat-based dish (≈23 mg/kg), shawarma (≈20 mg/kg) and minced cutlets (≈19 mg/kg). The high value for offal reflects the composition of “tzhvzhik”, which is usually prepared from animal liver, heart and lungs, i.e., organs that are among the richest animal sources of iron.

### 3.2. Probabilistic Assessment of Daily Fe Intake in the Armenian Diet Based on Individual Food Consumption Data (Single 24-h Recall)

Monte Carlo simulation (with 100,000 iterations) for the probabilistic assessment of dietary iron intake using individual food consumption data resulted in distributions of observed one-day intake for total iron, heme iron and non-heme iron (Figure 3, Figure 4 and Figure 5). Total dietary iron intake showed a right skewed distribution in all studied population groups, with the mean exceeding the median throughout (Figure 3). For men, the estimated mean daily intake was 10.39 mg/day (median of 9.61 mg/day), with a 5th–95th percentile range of 3.02–20.44 mg/day. Women aged 18–39 years had a mean intake of 8.31 mg/day (median of 7.61 mg/day), while women aged ≥40 years had a comparable mean intake of 8.26 mg/day (median of 7.40 mg/day). The corresponding 5th–95th percentile ranges were 1.77–17.26 and 1.77–17.67 mg/day, respectively. The P99 values were 24.52, 20.92 and 21.78 mg/day for men, women aged 18–39 years and women aged ≥40 years, respectively.

Non-heme iron represented the major contribution in total dietary iron intake across all study population groups (Figure 4). Mean daily non-heme iron intake was 8.90 mg/day for men, 7.48 mg/day for women aged 18–39 years and 7.49 mg/day for women aged ≥40 years. Median intakes were 8.31, 6.76 and 6.83 mg/day, respectively. The 95th percentile (P95) of non-heme iron intake reached 17.05 mg/day for men, 15.88 mg/day for women aged 18–39 years and 15.42 mg/day for women aged ≥40 years.

Heme iron contributed a smaller proportion of total dietary iron intake (Figure 5). Among participants who reported at least one heme iron source on the recall day (men, n = 445; women aged 18–39 years, n = 173; and women aged ≥40 years, n = 211), mean heme iron intake was 4.86, 4.98 and 2.80 mg/day, respectively. The corresponding median intakes were 4.28, 4.38 and 2.46 mg/day, while the 99th percentile values reached 12.82, 13.24 and 7.42 mg/day. These values are conditional on consumption and are therefore not directly comparable with the total and non-heme iron intake reported above, which refer to all participants of each group. The heme iron mean estimates represent intake among heme-iron consumers only, whereas total dietary iron intake was calculated for all participants. Therefore, the slightly higher mean heme iron intake among women aged 18–39 years in this subgroup does not contradict the higher total dietary iron intake estimated among men across the entire study population. It should be noted that these values are outputs of the triangular distribution fitted to heme iron intake. For a right-skewed variable such as heme iron intake (Figure 5), this gives greater weight to the upper end of the range than the PERT distribution fitted to total and non-heme iron intake. Therefore, the results can be interpreted as a characterization of the distribution of heme iron intake among its consumers and not as a directly comparable estimate of the average heme iron intake of the study population groups.

### 3.3. Deterministic Assessment of Daily Fe Intake in Armenian Diet Based on Household Budget Survey Data

The deterministic assessment based on household food consumption data from the Statistical Committee of Armenia indicated that in 2019–2024 per capita total daily intakes of dietary iron in the general population of Armenia (Supplementary Material, Table S12) varied between 8.07 and 9.27 mg/day, with the highest average value (9.27 mg/day) being recorded for 2024 (Figure 6). Overall, the estimated iron intake showed a gradual increasing trend during the investigated period. Since the same iron concentrations were applied to all years, this trend reflects changes in the reported household food consumption quantities only.

Different food groups’ assessment data analysis shows that a permanent source of dietary iron comprises flour and flour-based food products, the contribution of which constitutes the larger share in the total intake of iron and shows an increasing trend (approximately 1.2 times) from 2019 (3.9 mg/day) to 2024 (4.58 mg/day), reflecting changes in the reported per capita consumptions of these foods (Supplementary Table S13). The second food group that provides daily iron intake is meat and meat-based products, the contribution of which has also increased more than 1.3 times: from 1.41 mg/day (2019) to 1.87 mg/day (2024). This is relevant because meat, meat products and fish are the only sources of heme iron in the diet. The third food group includes fresh and processed vegetables, whose contribution to iron intake underwent no substantial dynamic changes in 2019–2024. In 2024, flour and flour-based foods provided approximately 50% of total estimated dietary iron intake, while meat and meat-containing products contributed 20% (Figure 7). The per capita consumption of the main food groups is presented in Supplementary Table S13. Since the iron concentrations in flour-based foods (Table S1) are comparable to or lower than those of most meat products (Table S9), the dominant contribution of flour-based foods is driven mainly by their high consumption rather than by high iron concentrations. It is important to note that mandatory iron fortification of wheat flour is not implemented in Armenia, and none of the sampled flour-based products carried a declaration of added iron (i.e., fortification).

## 4. Discussion

In Armenia, the iron content of different food groups showed considerable variations depending on food type, origin and processing (e.g., fresh, processed, including dried, canned products, etc.), consistent with the compositional characteristics reported in national [38] and international food composition databases [39,40]. For most foods, the iron concentrations obtained in the present study were within the range reported in these databases. Differences between datasets are expected, because TDS samples are analysed as consumed (i.e., after cooking), particularly as changes in water content during preparation can affect the measured iron concentrations. Direct food-level comparisons with the earlier Armenian assessment [17] is not possible because the food-specific iron concentrations were not reported in the available publication; only the estimated dietary iron intake results and their discussion were presented. The earlier assessment [17] was based on flame AAS and included a smaller number of food items, whereas the present study quantified iron concentrations using ICP-OES and included 146 composite samples representing a broader and more detailed range of foods, including composite dishes, analysed as consumed.

The contributions of individual foods or food groups to total dietary iron intake are determined not only by their iron content but also the quantity and frequency of consumption. Particularly, foods with the highest iron concentrations in this study (e.g., dried basil, bitter chocolate, ground black pepper, offal, etc.) are typically associated with small portion sizes or low consumption frequency, resulting in only a minor contribution to total dietary iron intake. In contrast, foods containing moderate amounts of iron may contribute more to total dietary iron intake than foods with higher iron content when consumed more frequently and in large portions. Accordingly, the present study showed that flour-based products were the major contributors to dietary iron intake in the Armenian population (Figure 7). Although the slight increase observed in the contribution of meat and meat-containing products between 2019 and 2024 (Figure 6) may represent a favourable change in the dietary pattern, their contribution remained considerably lower than that of flour-based products, indicating that the Armenian diet continues to rely predominantly on plant-origin sources of iron.

The Monte Carlo simulations further showed that non-heme iron represented the predominant fraction of total dietary iron intake in the studied population (Figure 4). This finding reflects the dietary characteristics of the Armenian population [17], where flour-based products, fresh and processed fruits and vegetables, potatoes and other plant-derived foods are the main contributors to dietary non-heme iron intake. Although heme iron (Figure 5) contributed a smaller proportion to total intake, it remains nutritionally significant because of its higher bioavailability compared with non-heme iron [41]. Plant-origin sources contain only non-heme iron; however, their high consumption frequency and quantity result in a considerable contribution to total iron intake. The predominance of non-heme iron emphasizes the importance of considering the dietary factors influencing iron bioavailability, including absorption promoters such as vitamin C and inhibitors such as phytates and polyphenols [42,43,44]. It should be emphasized, however, that the present study estimated dietary iron intake, i.e., the amount of iron ingested, and not the amount absorbed and physiologically available. The absorption of iron varies widely depending on meal composition and on the iron status of the individual [13,42] and was not modelled in this study. The considerations regarding enhancers and inhibitors therefore indicate the likely direction of their effect on bioavailability, but the calculated intakes should not be interpreted as absorbed iron. Algorithms for estimating absorbed iron could be applied in future studies once meal-level data on enhancers and inhibitors become available.

The probabilistic distribution of dietary iron intake within the Armenian adult population captured inter-individual variability in dietary intake among different groups. The estimated percentile values confirmed this variability. The median (P50) represents the intake below which half of the studied population falls and therefore characterizes the typical dietary iron intake of the Armenian adult population, whereas the upper percentiles (P75 and P95) describe progressively higher intake scenarios and identify individuals with relatively high dietary iron intakes. In this study, the consistently higher percentile values of total iron intake observed among men compared with women indicate that men consumed larger amounts of dietary iron across the entire intake distribution rather than only on average. Therefore, the probabilistic approach provided more information than mean intake estimates alone by identifying both lower- and higher-intake population groups that cannot be distinguished using deterministic average estimates.

Within the studied population groups, men had the highest mean total dietary iron intake (10.39 mg/day), while women aged 18–39 years and women aged ≥40 years had comparable mean intake (8.31 and 8.26 mg/day, respectively). However, interpretation of these values requires consideration of gender and age-specific physiological requirements. The estimated intakes were compared with the EFSA Dietary Reference Values (DRVs) for iron [1,45]. The EFSA DRVs were selected because no national DRVs for iron have been established in Armenia and because EFSA provides age- and sex-specific DRVs for adults (≥18 years), including separate values for men as well as premenopausal (18–39 years) and postmenopausal (≥40 years) women. Hence, participants in the 24 h dietary recall survey were grouped according to these EFSA age- and sex-specific groups, and the corresponding DRVs were applied to estimate dietary iron intake adequacy. According to the EFSA DRVs [1,45], the Average Requirement (AR) for iron is 6 mg/day for men and postmenopausal women and 7 mg/day for premenopausal women. The AR represents the daily iron intake estimated to meet the requirements of half of the healthy individuals in a population; therefore, it should not be interpreted as a recommended intake level for all individuals. In contrast, the Population Reference Intake (PRI) represents an intake level sufficient to meet the requirements of almost all of the healthy population, so it is considerably higher (11 mg/day for men and postmenopausal women and 16 mg/day for premenopausal women). The PRI was not used as a cut-off for inadequacy. In this study, the estimated mean intake for men (10.39 mg/day) exceeded the AR (6 mg/day). Similarly, the median intake (9.61 mg/day) was above the AR, suggesting that at least half of the male population had iron intakes exceeding the average dietary requirements. Applying the AR as a cut-off point, 19.8% of the simulated one-day intakes among men were below 6 mg/day. The mean and median values remained below the PRI of 11 mg/day; however, mean or median intakes below the PRI do not in themselves indicate inadequate intake or inadequate nutritional status, because the PRI is not a cut-off for assessing the prevalence of inadequacy in groups. The prevalence of inadequate intake in a group is better approximated by the proportion of intakes below the AR. Evaluation of the upper end of the intake distribution showed that higher percentiles (i.e., P95 and P99) approached or exceeded the PRI, while the lower percentiles (e.g., P5) were below the AR. A similar pattern was observed among women aged ≥40 years. The mean and median dietary intakes (8.26 and 7.40 mg/day, respectively) exceeded the AR of 6 mg/day; they remained below the PRI of 11 mg/day. In this group, 36.1% of the simulated one-day intakes were below the AR of 6 mg/day, i.e., a higher proportion than among men. Premenopausal women showed the largest gap between intake and the PRI. Although the mean intake (8.31 mg/day) and median intake (7.61 mg/day) exceeded the AR of 7 mg/day, they remained substantially below the PRI of 16 mg/day. The proportion of intakes below the AR was also the highest in this group: 47.6% of the simulated one-day intakes were below the AR of 7 mg/day, more than twice the proportion observed among men. However, the estimate for this group should be interpreted with caution, since two opposite biases act on it: the AR cut-off point approach is not strictly valid and tends to underestimate the prevalence of inadequate intake, whereas the use of one-day rather than habitual intake data widens the intake distribution and tends to overestimate it. A probability approach applied to usual-intake distributions would be required for a more precise estimate. Importantly, comparison of the highest percentile (P99) intake value (20.92 mg/day) with the PRI provides additional perspective, as high consumers approaching the upper tail of the intake distribution may only reach the intake level for almost all individuals, highlighting the challenge of achieving sufficient dietary iron intake among the group of premenopausal women. These findings suggest that although mean and median intakes exceed the AR at the population level, inadequate dietary iron intake remains a potential nutritional concern for vulnerable subgroups, particularly women of reproductive age [17]. Whether this translates into iron deficiency cannot be established from intake data alone and requires biomarker assessment.

The present findings are generally consistent with international evidence indicating that inadequate dietary iron intake remains one of the most common micronutrient concerns worldwide, particularly among women of reproductive age [46,47,48,49,50]. In addition, the predominance of flour-based foods as the main source of dietary iron is not unique to Armenia. Cereal products are major contributions to iron intake in many populations with mixed diets, providing 31.3% of iron intake in Italy, where meat products contribute 16.9% [51], 34.1% in Poland according to household budget survey data [52], and 29.7% among American adults [53]. In Slovenia, bread and bakery products, meat, fruit and vegetables were reported as the main dietary sources of iron [54]. The share observed in Armenia (≈50% in 2024) is, however, considerably higher, reflecting the central role of bread, lavash and other flour-based products in the Armenian diet [19,23]. From a public health perspective, the estimated mean total dietary iron intake of the Armenian adult population (probabilistic assessment, Figure 3) and the general population (deterministic assessment, Figure 6) remained below the Eurasian Economic Union (EAEU) reference value of 14 mg/day, which is intended for food labelling purposes in EAEU member countries, including Armenia [55]. Although this reference value is not intended to assess nutritional adequacy, it provides additional regulatory context regarding the overall level of dietary iron intake in the Armenian population and may be useful for evaluating the potential contribution of iron-fortified foods and nutrition claims.

The upper end of the intake distributions (Figure 3) was also compared with the safe level of iron intake of 40 mg/day [45,56]. Because the available data were insufficient to derive a tolerable upper intake level (UL), EFSA established this safe level as the highest level of daily intake of iron from all sources for which there is reasonable confidence regarding the absence of adverse effects [56]. The maximum values of a Monte Carlo simulation depend on the model and on the number of iterations and do not represent the maximum intake in the population; therefore, upper percentiles were used for this comparison. The P95 (20.44, 17.26 and 17.67 mg/day) and P99 (24.52, 20.92 and 21.78 mg/day) for men, women aged 18–39 years and women aged ≥40 years, respectively, were well below 40 mg/day. These findings suggest that excessive iron intake from the diet alone is unlikely in the Armenian adult population under current dietary conditions. However, iron from dietary supplements was not captured, and population-level reference values do not protect individuals with a predisposition to iron overload [56].

Overall, the main strengths of the study are the analytical determination of iron in 146 TDS composite samples of foods prepared as consumed and the combined use of individual and household food consumption data. Several limitations, however, should be considered. First, the individual assessment is based on a single 24 h recall per participant; thus, the results describe observed one-day intake rather than habitual intake, the tails of the distributions are wider than those of habitual intake, and the proportions below the AR are likely to be overestimated. Future national surveys should include at least a second, non-consecutive recall in a subsample. Second, the household ILCS data refer to food purchased and consumed at the household level; they do not account for food waste, foods consumed away from home or the distribution of food among household members, and they are expressed per capita across ages. They are therefore suitable for monitoring trends and identifying the main dietary sources but not for estimating intake distributions. Third, the estimation of heme iron relied on the assumption that 40% of iron in meat and meat products, poultry and fish is heme iron. This fixed factor may underestimate the heme iron content of red meat and overestimate it in poultry, fish and well-cooked composite dishes. However, this assumption affects only the estimated heme/non-heme split and does not affect total dietary iron intake. Fourth, data on dietary supplement use were not collected. In addition, the Goldberg cut-off was not applied in this study, since energy intake was not computed from the recall records; therefore, the plausibility of the reported intakes could not be evaluated against energy requirements, and implausible low energy reports could not be identified. As under-reporting of food intake is common in 24 h dietary recall, dietary iron intake may consequently be underestimated, and the proportion of intakes below the AR correspondingly overestimated. Fifth, age ≥40 years was used as a proxy for postmenopausal status consistent with the EFSA DRV database [45], because menopausal status was not recorded for female participants in the 24 h dietary recall survey. A proportion of postmenopausal women (e.g., aged 40–49 years) are likely to be still menstruating, which may lead to an underestimation of dietary iron intake inadequacy when age ≥40 years is used as a proxy for postmenopausal status. Finally, no biochemical indicators of iron status (e.g., haemoglobin, serum ferritin or transferrin saturation) were measured. Therefore, the results characterize dietary iron intake in relation to DRVs and cannot be used to infer iron status or the prevalence of iron deficiency or anaemia.

## 5. Conclusions

This study provides a comprehensive assessment of dietary iron intake in Armenia by integrating a deterministic household-based approach with a probabilistic individual-based approach using Monte Carlo simulation. The use of household food consumption data from the Armenian Integrated Living Conditions Survey over a six-year period (2019–2024) enabled the assessment of temporal variability in population-level iron intake that a single-time-point 24 h recall cannot resolve. Although household-level data do not account for individual food distribution within households, food waste or foods consumed outside the home, they represent a valuable and regularly updated source of dietary information for monitoring population-level nutrient intake, particularly in settings where nationally representative individual dietary surveys (e.g., 24 h recall survey) are limited.

The integrated assessment showed that deterministic estimates are valuable for identifying major dietary contributions and evaluating trends over time, whereas probabilistic modelling provides additional information on the distribution of single-day iron intake and the proportion of intakes below the AR. The estimated average dietary iron intakes exceeded the EFSA AR (6 mg/day for men and postmenopausal (≥40 years) women, and 7 mg/day for premenopausal (18–39 years) women), indicating adequate intake levels for a substantial proportion of the population, while 19.8% of men, 47.6% of premenopausal women and 36.1% of postmenopausal women had one-day intakes below the AR. However they remained below the PRI (11 mg/day for men and postmenopausal women and 16 mg/day for premenopausal women), with the largest gap among women aged 18–39 years. These findings indicate a potential risk of inadequate dietary iron intake in vulnerable population groups but do not by themselves demonstrate inadequate iron status, which requires biomarker assessment. Therefore, linking dietary intake data with biomarker data in future national surveys is a priority.

Overall, the findings indicate that addressing potentially inadequate dietary iron intake in Armenia requires targeted and specific strategies rather than a uniform population-wide approach. The study outcomes support the development of evidence-based public health initiatives and dietary interventions tailored to population groups with increased nutritional risk, particularly women of reproductive age. Meanwhile, the upper percentiles of dietary iron intake were below the EFSA safe level of 40 mg/day, suggesting that excessive iron intake from the usual diet is unlikely under the current dietary conditions in Armenia, although supplements were not considered.

## Figures and Tables

**Figure 1:**
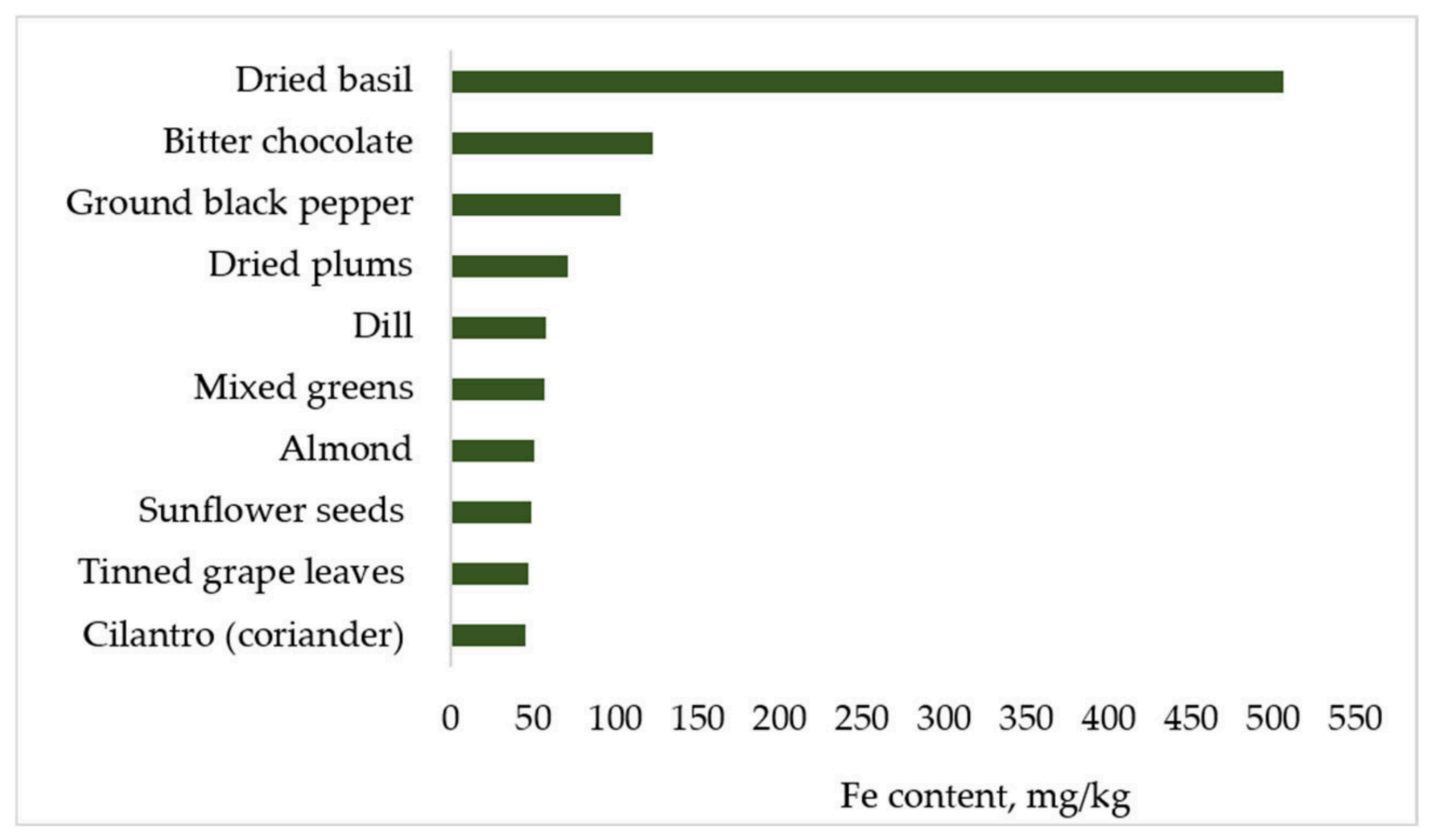
Top ten plant-origin products highest in iron (Fe) in the Armenian diet (mean values for one composite sample per food).

**Figure 2:**
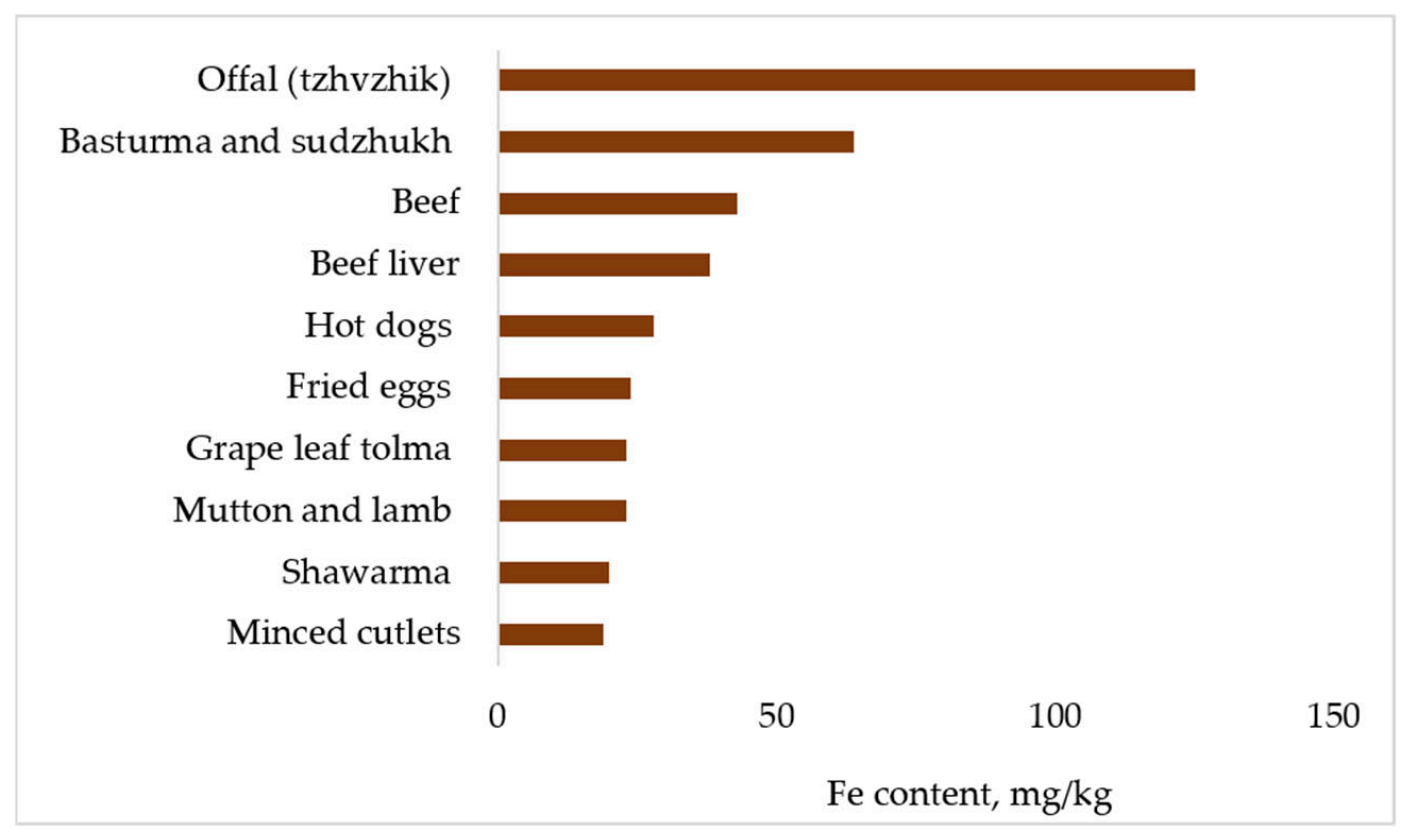
Top ten animal-origin products (and animal product-based foods) highest in iron (Fe) in the Armenian diet (mean values for one composite sample per food).

**Figure 3:**
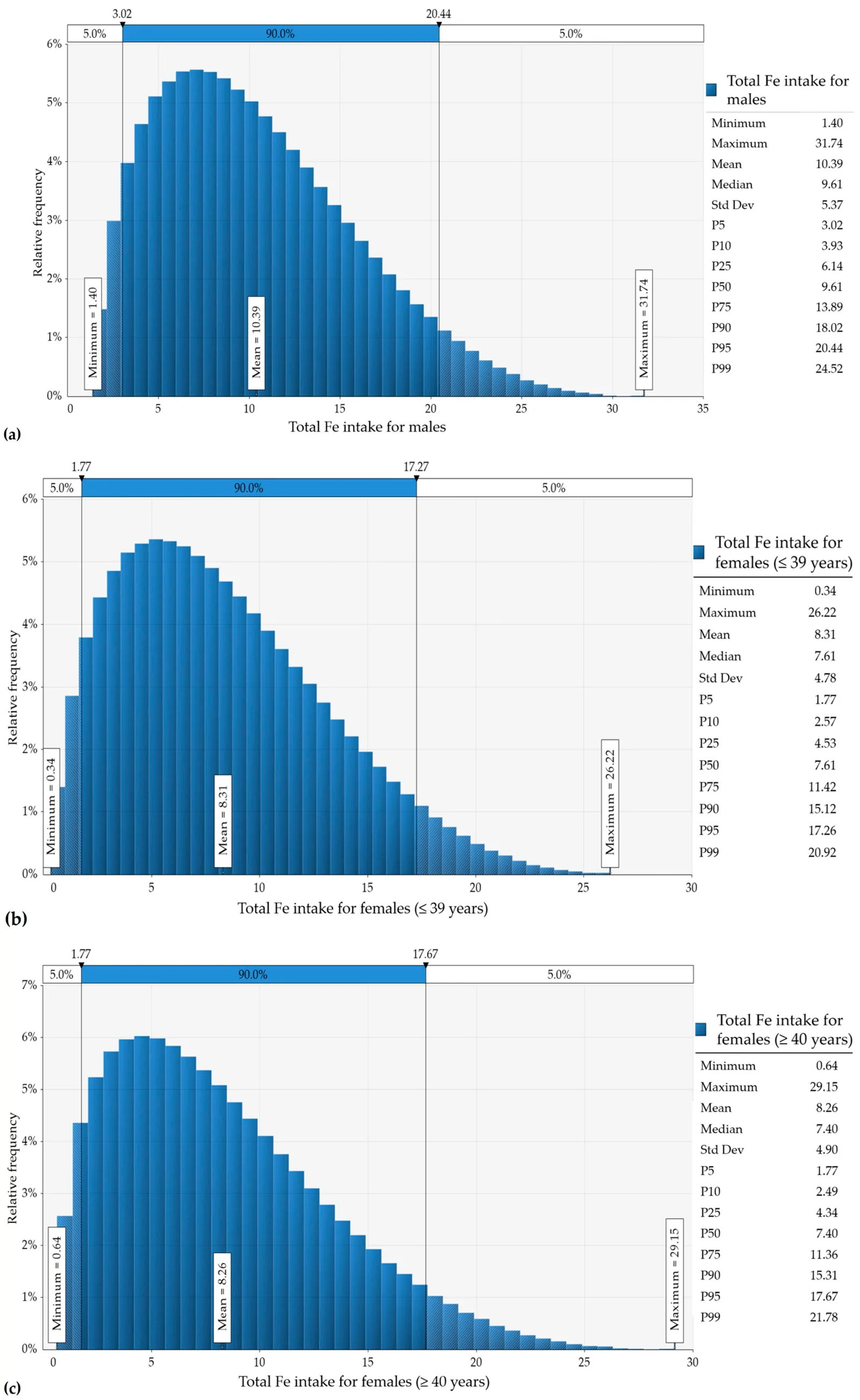
Probabilistic distribution of total dietary iron (Fe) intake among studied population: men (**a**), women aged 18–39 years (**b**), and women aged ≥40 years (**c**).

**Figure 4:**
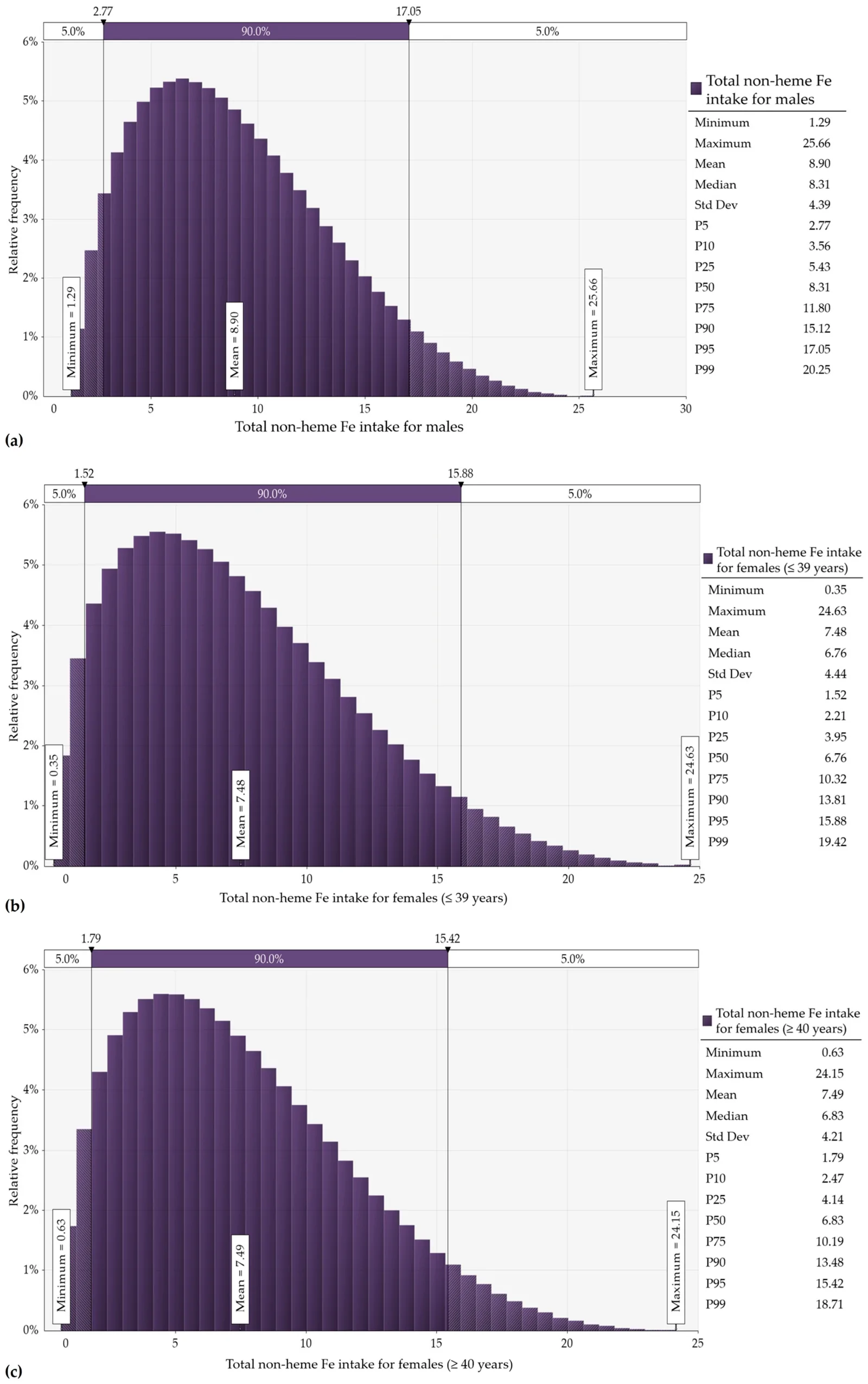
Probabilistic distribution of dietary non-heme iron (Fe) intake among studied population: men (**a**), women aged 18–39 years (**b**), and women aged ≥40 years (**c**).

**Figure 5:**
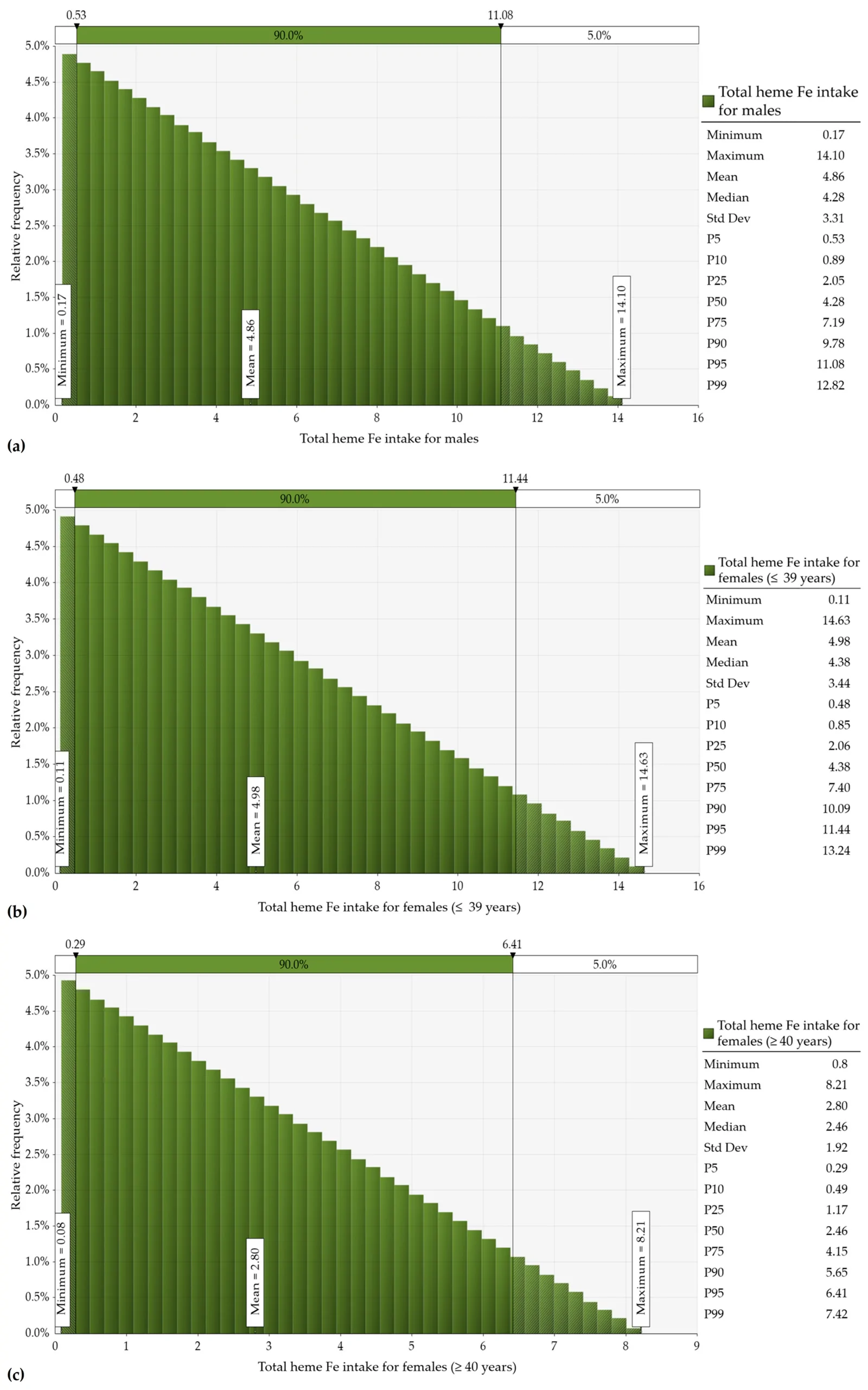
Probabilistic distribution of dietary heme iron (Fe) intake among studied population: men (**a**), women aged 18–39 years (**b**), and women aged ≥40 years (**c**).

**Figure 6:**
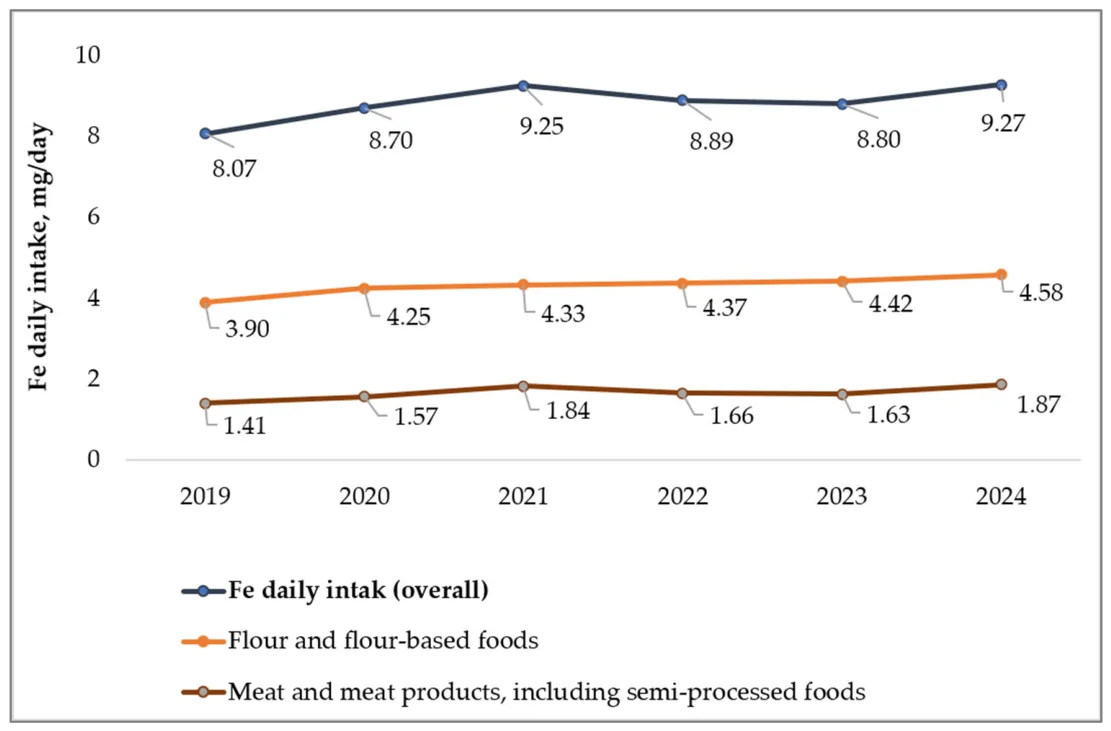
Daily intake of iron based on the household food consumption data for 2019–2024 (ARMSTAT, 2026).

**Figure 7:**
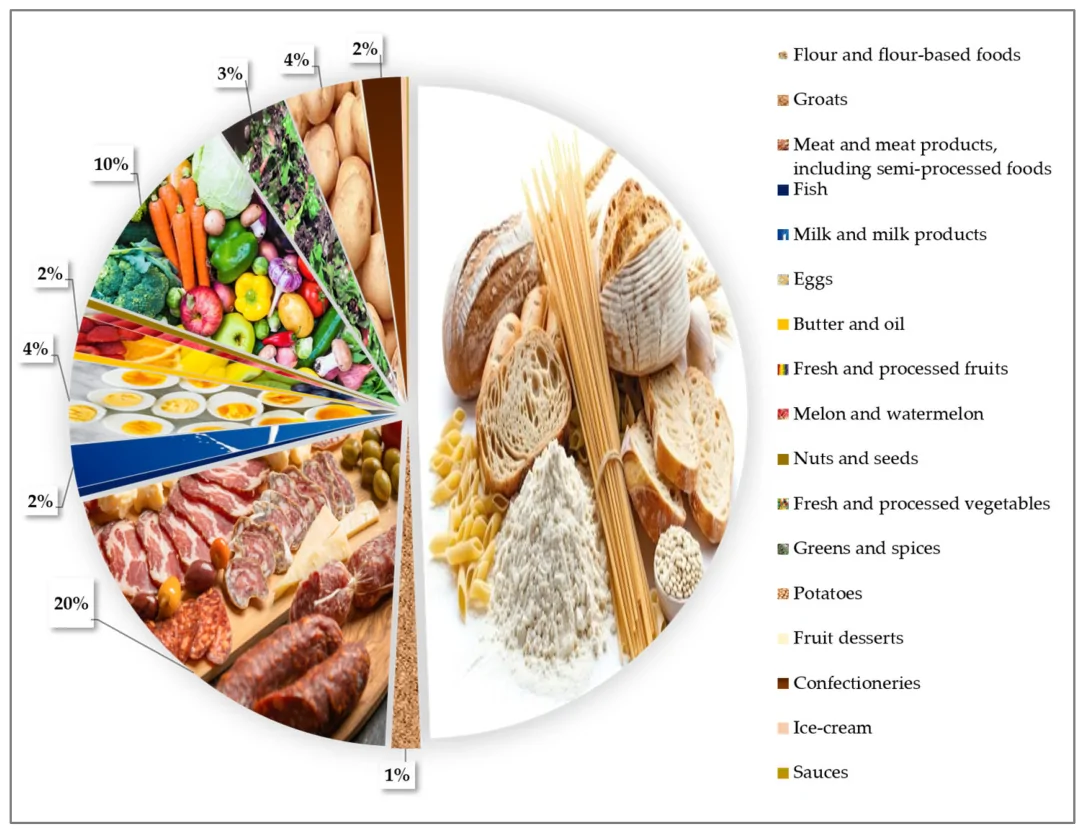
The contribution of different food groups to daily iron intake (using the example of 2024).

## Data Availability

The original contributions presented in the study are included in the article/Supplementary Materials. Further inquiries can be directed to the corresponding author.

## Supplementary Materials

The following supporting information can be downloaded at https://www.mdpi.com/article/10.3390/foods15213868/s1. Table S1. Iron (Fe) content in flour and flour-based products. Table S2. Iron (Fe) content in grocery products. Table S3. Iron (Fe) content in fresh and processed vegetables. Table S4. Iron (Fe) content in greens and spices. Table S5. Iron (Fe) content in fresh and processed fruits. Table S6. Iron (Fe) content in fruit confectionery, chocolates and ice cream. Table S7. Iron (Fe) content in nuts and seeds. Table S8. Iron (Fe) content in milk and milk products. Table S9. Iron (Fe) content in meat and meat products, fish and eggs. Table S10. Iron (Fe) content in fat and oil products. Table S11. Iron (Fe) content in other products. Table S12. Daily iron intake in the Armenian population diet based on the household food consumption data (2019–2024). Table S13. Per capita daily mean consumption of food products by the Armenian population based on household food consumption data (g/day) (2019–01；2024). Table S14. Quality control of iron (Fe) determination by ICP-OES. Table S15. Repeatability of iron (Fe) determination in independently re-digested composite samples. Table S16. Variables, fitted probability distributions and output statistics of the Monte Carlo model of one-day dietary iron intake. References [57,58] are cited in Supplementary Materials.

## Abbreviations

The following abbreviations are used in this manuscript:

AAS	Atomic Absorption Spectroscopy
AR	Average Requirement
CENS	Center for Ecological-Noosphere Studies
DI	Daily Intake
DNA	Deoxyribonucleic Acid
DRV	Dietary Reference Value
EAEU	Eurasian Economic Union
EFSA	European Food Safety Authority
Fe	Iron
HBSs	Household Budget Surveys
ICP-OES	Inductively Coupled Plasma Optical Emission Spectroscopy
IDA	Iron Deficiency Anaemia
ILCS	Integrated Living Conditions Survey
LOD	Limit of Detection
LOQ	Limit of Quantification
P5	5th percentile
P10	10th percentile
P25	25th percentile
P50	50th percentile
P75	75th percentile
P90	90th percentile
P95	95th percentile
P99	99th percentile
PRI	Population Reference Intake
QA/QC	Quality Assurance and Quality Control
TDS	Total Diet Study
UL	Tolerable Upper Intake Level
WHO	World Health Organization
