# Supplementary material for: Probabilistic and Deterministic Assessment of Dietary Iron Intake for Armenian Adult Population Using Individual and Household-Based Approaches
Source: Foods. 2026 Oct 29;15(21):3868. doi: 10.3390/foods15213868 (PMC13762536; doi:10.3390/foods15213868)
Supplement: Supplementary file 1 [file foods-15-03868-s001.zip › foods-4556251-supplementary.pdf]

Supplementary Material

Table S1. Iron (Fe) content in flour and flour-based products

Food group	Food name	Fe content (mg/kg)
**Flour**	Flour (wholegrain)	35.834
	High grade flour (called "Makfa")	7.682
	High grade flour (called "Ryazanochka")	7.666
	High grade flour (called "Baghramyan")	5.136
**Bread and lavash***	Bread (wholegrain and fitness)	24.486
	Rye bread	21.203
	Lavash	18.279
	Bread from high grade flour	16.865
	Bread (called "Matnaqash")	9.896
	Bread (called "Hrazdan")	7.917
**Bakery**	Waffles	34.899
	Biscuits/cookies	16.237
	Non-cream cake	13.102
	Cream cake	11.729
	Gata	6.880
**Other bakery products**	Salted sticks	16.261
	Crackers	11.820

Note: *Lavash is a traditional thin flatbread. Gata is a traditional sweet pastry.*

Table S2. Iron (Fe) content in grocery products

Food group	Food name	Fe content (mg/kg)
**Pasta products**	Pasta	9.042
	Spaghetti	7.529
	Vermicelli	6.786
**Cereal grains**	Emmer	11.059
	Buckwheat	9.389
	Rice	2.858
**Legumes and pulses**	Dry beans	28.374
	Lentils	26.038
	Chickpeas	21.933

Table S3. Iron (Fe) content in fresh and processed vegetables

Food group	Food name	Fe content (mg/kg)
**Fresh vegetables**	Tomato	1.827
	Cucumber	5.181
	Pepper	1.241
	Carrot	1.408
	Beetroot	4.559
	Zucchini	4.934
	Eggplant	1.688
	Cabbage	2.322
	Cauliflower	1.898
	Broccoli	3.436
	Green beans	6.588
	Garlic	5.504
	Onion	0.347
**Potato**	Potato (fried)	6.199
	Potato (boiled, prepared with butter	0.525
**Canned vegetables**	Tinned grape leaves	47.025
	Tomato paste	28.481
	Green peas	19.531
	Pickled cucumbers	13.529
	Pixeled vegetables	7.009
	Mushrooms	5.525

Table S4. Iron (Fe) content in greens and spices

Food group	Food name	Fe content (mg/kg)
**Fresh greens (herbs)**	Dill	58.528
	Mixed greens (herbs)	56.551
	Cilantro (coriander leaves)	45.415
**Spices**	Dried basil	506.058
	Ground black pepper	102.883
	Table salt	0.891

Table S5. Iron (Fe) content in fresh and processed fruits

Food group	Food name	Fe content (mg/kg)
**Pome fruits**	Quince	2.487
	Pear	0.749
	Pomegranate	0.430
	Apple	n/d (<0.0004)
**Stone fruits**	Plum	5.127
	Apricot	3.845
	Sweet cherry	3.753
	Grape	2.177
	Sour cherry	1.451
	Peach	n/d (<0.0004)
	Nectarine	1.261
**Tropical and subtropical fruits**	Dates	14.059
	Fig	5.491
	Persimmon	3.008
	Banana	1.863
**Citrus fruits**	Kiwi fruit	0.848
	Lemon	0.656
	Mandarin	0.145
	Orange	n/d (<0.0004)
	Grapefruit	n/d (<0.0004)
**Berries**	Raspberry	6.574
	Strawberry	2.180
**Melons**	Melon	1.615
	Watermelon	0.927
**Dried fruits**	Dried plums	70.590
	Dried apricots	39.468
	Mixed dried fruits	2.571

Note: *n/d – not detected, i.e., <LOD (limit of detection).*

Table S6. Iron (Fe) content in fruit confectionery, chocolates and ice cream

Food group	Food name	Fe content (mg/kg)
**Fruit confectionery**	Sweet sudzhukh	15.264
	Fruit jam	4.076
**Chocolates**	Bitter chocolate	122.998
	Milk chocolate	36.864
**Ice cream***	Ice-cream	9.038

Note: *Sweet sudzhukh is a fruit and walnut confection. * - Although ice cream is generally classified as a dairy product, it is presented as a separate food group following the confectionery products category in the microdata database of the Statistical Committee of the Republic of Armenia.*

Table S7. Iron (Fe) content in nuts and seeds

Food group	Food name	Fe content (mg/kg)
**Nuts**	Almonds	51.067
	Hazelnuts	43.237
	Walnuts	26.891
	Cashew nuts	25.864
	Peanuts	21.659
**Seeds**	Sunflower seeds	49.057

Table S8. Iron (Fe) content in milk and milk products

Food name	Fe content (mg/kg)
Cheese products	7.186
Cheese	5.412
Curd/cottage cheese	5.610
Kefir	2.725
Yogurt	2.229
Sour cream product	2.529
Sour cream	1.492
Matsun	1.752
Tan	0.975
Pasteurized milk	0.975

Table S9. Iron (Fe) content in meat and meat products, fish and eggs

Food name	Fe content (mg/kg)
Beef	42.685
Mutton and lamb	23.167
Pork	12.802
Chicken	7.620
Basturma and sudzhukh	63.875
Minced cutlets	18.822
Kebab	14.648
Sausage products	14.770
Cooked sausage	13.298
Kufta (meat containing culinary product)	10.444
Fillet and pashina	9.091
Offal (tzhvzhik)	125.324
Beef liver	38.140
Fish	5.722
Fried eggs	24.035
Boiled eggs	18.247

Table S10. Iron (Fe) content in fat and oil products

Food name	Fe content (mg/kg)
Butter	2.962
Fat and vegetable oil	7.665
Spread	6.400
Vegetable oil	0.725

Table S11. Iron (Fe) content in other products

Food group	Food name	Fe content (mg/kg)
**Pies**	Potato pie	7.937
	Hot dogs	28.126
**Products with dough and meat**	Pizza	13.302
	Lahmajo	15.763
**Meat dishes, soup and salad**	Khinkali	11.938
	Pelmeni	9.666
	Shawarma	19.66
	Grape leaf tolma	23.142
	Cabbage leaf tolma	12.901
	Harisa	2.119
	Spas	1.978
	Tabbouleh	17.544
**Sauces**	Mayonnaise	9.531
	Ketchup	7.436
**Coffee**	Black coffee (boiled)	1.300

Note: Pelmeni is a meat dumpling, common in the cuisine of Russia and post-Soviet countries. Khinkali is an herb and meat-filled dumpling, commonly made in Georgia and other Caucasian countries, including Armenia. Harisa is traditional pilov with emmer. Spas is a traditional soup prepared with matsun and groat.

Table S12. Daily iron intake in the Armenian population diet based on the household food consumption data (2019-2024)

Food groups	Daily iron intake (DI, mg/day) for 2019-2024 period					
	2019	*2020*	*2021*	*2022*	*2023*	*2024*
Flour and flour-based foods	3.899	4.251	4.330	4.368	4.425	4.578
Groats	0.128	0.138	0.145	0.123	0.133	0.132
Meat and meat products, including semi-processed foods	1.410	1.574	1.836	1.655	1.635	1.866
Fish	0.047	0.040	0.043	0.049	0.039	0.039
Milk and milk products	0.189	0.192	0.203	0.188	0.170	0.194
Eggs	0.371	0.378	0.378	0.404	0.406	0.378
Butter and oil	0.052	0.056	0.053	0.050	0.044	0.031
Fresh and processed fruits	0.133	0.130	0.185	0.174	0.152	0.163
Watermelon and melon	0.042	0.038	0.038	0.033	0.031	0.042
Nuts and seeds	0.016	0.018	0.057	0.032	0.031	0.035
Fresh and processed vegetables	0.961	1.062	1.107	0.978	0.952	0.944
Greens and spices	0.276	0.305	0.283	0.311	0.283	0.315
Potatoes	0.347	0.353	0.371	0.353	0.329	0.338
Fruit desserts	0.005	0.007	0.010	0.006	0.005	0.006
Confectioneries	0.165	0.140	0.176	0.142	0.140	0.176
Ice cream	0.024	0.020	0.027	0.021	0.020	0.023
Sauces	0.002	0.002	0.005	0.004	0.004	0.007
$\sum DI$	***8.07***	***8.70***	***9.25***	***8.89***	***8.80***	***9.27***

Note: *Flour-based products include bread, lavash, bakery products and pasta. "Meat and meat products, including semi-processed foods" group includes beef, pork, chicken, mutton and lamb, sausages, offal, kebab, minced cutlets, pelmeni, and khinkali.*

Table S13. Per capita daily mean consumption of food products by the Armenian population based on household food consumption data (g/day) (2019-2024)

Food groups	Food consumption (g/day) for 2019-2024 period					
	2019	*2020*	*2021*	*2022*	*2023*	*2024*
Flour and flour-based foods	256.493	275.342	280.306	284.966	286.243	293.497
Groats	21.037	21.953	23.228	20.616	24.031	23.852
Meat and meat products, including semi-processed foods	64.502	73.275	81.200	73.527	74.409	86.474
Fish	8.263	7.076	7.478	8.489	6.738	6.826
Milk and milk products	63.822	65.336	69.405	63.157	56.894	64.293
Eggs	17.536	17.880	17.902	19.110	19.182	17.886
Butter and oil	19.237	22.141	22.237	19.517	18.754	17.530
Fresh and processed fruits	38.020	41.970	50.405	44.544	44.370	48.967
Watermelon and melon	41.559	38.220	36.937	32.061	30.432	42.059
Nuts and seeds	0.447	0.487	1.755	0.965	0.930	1.056
Fresh and processed vegetables	140.984	156.438	160.016	144.613	139.881	151.803
Greens and spices	5.240	5.786	5.386	5.909	5.353	5.970
Potatoes	103.158	104.914	110.244	104.976	97.978	100.681
Fruit desserts	1.161	1.697	2.141	1.415	1.187	1.230
Confectioneries	2.066	1.747	2.206	1.778	1.755	2.197
Ice cream	2.625	2.158	2.960	2.317	2.265	2.566
Sauces	0.260	0.273	0.520	0.481	0.489	0.783

Note: *Flour-based products include bread, lavash, bakery products and pasta. "Meat and meat products, including semi-processed foods" group includes beef, pork, chicken, mutton and lamb, sausages, offal, kebab, minced cutlets, pelmeni, and khinkali.*

Table S14. Quality control of iron (Fe) determination by ICP-OES

Material / sample type	n	Certified or added value	Measured value, mean ± SD (mg/kg)	Recovery, mean ± SD (%)	Recovery range (%)
CRM BCR-679, White cabbage (elements)	3	55 mg/kg	45.70 ± 6.62	83.1 ± 12.0	74.4 – 96.8
CRM NCS ZC73031, Carrot	2	148 mg/kg	110.58 ± 7.32	74.7 ± 4.9	71.2–78.2
Spiked food samples	8	0.05 mg Fe per sample	–	98.6 ± 7.5	88.4–111.2
Reagent blanks	1 per analytical batch	–	not detected (<LOD)	–	–

Note: SRM – standard reference material. Each composite sample was digested and measured with three replicate injections. Reagent blanks were carried through the complete digestion procedure with each analytical batch and iron was not detected in any of them (LOD = 0.0077 mg/kg; LOQ = 0.0230 mg/kg, fresh weight); blank correction was therefore not required.

Table S15. Repeatability of iron (Fe) determination in independently re-digested composite samples

Food sample	n	Individual determinations (mg/kg)	Mean ± SD (mg/kg)	RSD (%)
Basturma and sudzhukh	2	61.58; 66.17	63.87 ± 3.25	5.1
Pasta	2	10.16; 7.92	9.04 ± 1.58	17.5
Biscuit, non-cream	2	13.72; 12.49	13.10 ± 0.87	6.6
Beans	2	28.99; 27.76	28.37 ± 0.87	3.1
Dried apricots	2	44.98; 33.96	39.47 ± 7.80	19.8
Walnut	2	27.62; 26.16	26.89 ± 1.04	3.8
Egg, boiled	2	18.61; 17.88	18.25 ± 0.51	2.8
Mayonnaise	2	11.75; 7.31	9.53 ± 3.14	33.0
Bitter chocolate	3	140.34; 120.44; 108.78	123.18 ± 15.96	13.0
Lahmajo	2	21.30; 10.22	15.76 ± 7.83	49.7
Tabbouleh	2	16.72; 18.37	17.54 ± 1.16	6.6
Offal (tzhvzhik)	2	121.57; 129.08	125.32 ± 5.31	4.2
Tomato	2	1.88; 1.77	1.83 ± 0.08	4.5
Mixed greens	2	53.44; 59.66	56.55 ± 4.40	7.8
Raspberry	2	6.30; 6.85	6.57 ± 0.38	5.8
Green beans	2	6.38; 6.80	6.59 ± 0.29	4.5
Broccoli	2	3.64; 3.23	3.44 ± 0.29	8.4
Melon	2	1.64; 1.59	1.62 ± 0.04	2.2
Beef liver	2	35.92; 40.36	38.14 ± 3.14	8.2
Cabbage	2	2.67; 1.97	2.32 ± 0.49	21.3
Grapes	2	1.88; 2.48	2.18 ± 0.42	19.5
Milk chocolate	2	22.35; 23.57	22.96 ± 0.86	3.7
Cabbage leaf tolma	2	12.51; 13.29	12.90 ± 0.55	4.3
High grade flour (called "Baghramyan")	2	4.70; 5.57	5.14 ± 0.61	12.0

Note: n – number of independent digestions of the same composite sample, each measured with three replicate injections. RSD – relative standard deviation between independent digestions. Repeatability was additionally assessed by re-digesting and re-analyzing 24 of the 146 composite samples in an independent analytical batch consistent with the use of replicate analyses as a routine quality control approach for assessing analytical precision in food analysis (Kumssa, D. B., et al., 2022, https://doi.org/10.1038/s41597-022-01500-5; Millour, S., et al., 2010, https://doi.org/10.1007/s00769-010-0671-z).

- Kumssa, D. B., Mossa, A. W., Amede, T., Ander, E. L., Bailey, E. H., Botoman, L., Chagumaira, C., Chimungu, J. G., Davis, K., Gameda, S., Haefele, S. M., Hailu, K., Joy, E. J. M., Lark, R. M., Ligowe, I. S., McGrath, S. P., Milne, A., Muleya, P., Munthali, M., et al. Cereal grain mineral micronutrient and soil chemistry data from GeoNutrition surveys in Ethiopia and Malawi. *Scientific Data* **2022**, 9(1). https://doi.org/10.1038/s41597-022-01500-5
- Millour, S., Noël, L., Chekri, R., Vastel, C., Kadar, A., Guérin, T. Internal quality controls applied in inductively coupled plasma mass spectrometry multi-elemental analysis in the second French Total Diet Study. *Accreditation and Quality Assurance* **2010**, 15(9), 503–513. https://doi.org/10.1007/s00769-010-0671-z

Table S16. Variables, fitted probability distributions and output statistics of the Monte Carlo model of one-day dietary iron intake

Model component / statistic	Men	Women aged 18-39 years	Women aged ≥40 years
**Simulation settings**			
Software	@RISK (Lumivero's DecisionTools Suite)	@RISK (Lumivero's DecisionTools Suite)	@RISK (Lumivero's DecisionTools Suite)
Number of iterations	100,000	100,000	100,000
Participants included (n)	666	313	421
Criterion used for ranking candidate distributions	AIC	AIC	AIC
**Total dietary iron intake (mg/day), all participants**			
Fitted distribution	PERT	PERT	PERT
Goodness-of-fit statistic: AIC	3,969.5265	1,784.1485	2,346.2911
Mean	10.39	8.31	8.26
P5	3.02	1.77	1.77
P50	9.61	7.61	7.40
P95	20.44	17.26	17.67
P99	24.52	20.92	21.78
Intakes below the AR (%)	19.8	47.6	36.1
**Non-heme iron intake (mg/day), all participants**			
Fitted distribution	PERT	PERT	PERT
Goodness-of-fit statistic: AIC	3,710.0172	1,703.5945	2,241.4580
Mean	8.90	7.48	7.49
P50	8.31	6.76	6.83
P95	17.05	15.88	15.42
P99	20.25	19.42	18.71
**Heme iron intake (mg/day), consumers of heme iron sources only**			
Participants reporting heme iron sources (n)	445	173	211
Fitted distribution	Triangular	Triangular	Triangular
Goodness-of-fit statistic: AIC	1,905.7186	744.4753	677.0148
Mean	4.86	4.98	2.80
P50	4.28	4.38	2.46
P95	11.08	11.44	6.41
P99	12.82	13.24	7.42

Note: Distributions were fitted with the @RISK "Fit" tool, and the first ranked distribution was selected for each dataset. Candidate distributions were ranked by the Akaike information criterion (AIC) within each dataset; AIC values serve for the relative comparison of candidate distributions fitted to the same dataset and are not comparable between datasets of different size or between different variables. Total and non-heme iron intake statistics refer to all participants of each group, whereas heme iron intake statistics refer only to participants who reported at least one heme iron source on the recall day; the three fitted outputs are therefore not additive, and the heme iron statistics describe the intake distribution among consumers of heme iron sources and are not an additive component of total dietary iron intake. AR: Average Requirement (6 mg/day for men and women aged ≥40 years; 7 mg/day for women aged 18-39 years).
